# Supplementary material for: Barriers to and Facilitators of Adherence to Clinical Practice Guidelines in the Middle East and North Africa Region: A Systematic Review
Source: Healthcare (Basel). 2020 Dec 15;8(4):564. doi: 10.3390/healthcare8040564 (PMC7765264; doi:10.3390/healthcare8040564)
Supplement: Supplementary file 1 [file healthcare-08-00564-s001.pdf]

Supplementary materials

Table S1: Search Strategy

Generated using PubMed Advanced Search Builder	((CPG*) OR Clinical Practice Guideline*) OR Clinical guideline*) OR Guideline*) OR Practice Guideline*) AND Middle East*) OR Algeria*) OR Bahrain*) OR Kingdom of Bahrain) OR Djibouti) OR Egypt*) OR Arab Republic of Egypt) OR Iran*) OR Islamic Republic of Iran) OR Iraq*) OR Syrian Arab Republic) OR Syria*) OR Kuwait*) OR Hashemite Kingdom of Jordan) OR Jordan) OR Leban*) OR Lebanon) OR Morocco) OR Maghreb) OR Maghrib) OR Libya) OR Qatar) OR Somalia*) OR Sudan) OR Tunisia) OR Tunis) OR Saudi Arabia*) OR Kingdom of Saudi Arabia) OR Saudi) OR Yemen) OR United Arab Emirates) OR Emirat*) OR Palestine) OR Occupied Palestinian Territory) OR Israel) OR Gaza) OR Gazah) OR Gaza and the West Bank) OR The West Bank) OR Gulf Cooperation Council) OR Gulf) OR GCC

Table S 2. Barriers for implementations of clinical practice guidelines

Category	Barriers	Qualitative			Mixed		Quantitative								Total number
		Radwan	Alkhatib	Seyed	Wahbi	Elsadig	Radwan	Salma	Sharif	Jradi	Thabet	AlSubai	Alnoush	Al-Azzam	
Healthcare professional	Lack of awareness of the existence of guidelines				✓							✓			2
	Lack of familiarity with CPGs recommendation				✓							✓		✓	3
	Disagreement to the recommendations of the CPG				✓		✓					✓			3
	Doubts about the positive impact of CPGs on outcomes									✓			✓		2
	Preference for experience over CPGs			✓											1
	Lack of effective communication, research and self-learning skills			✓											1
	Lack of healthcare professional motivation			✓			✓					✓			3
Guidelines	Lack of clarity or complexity			✓			✓		✓			✓			4
	Rigid guidelines may not always be practical and cannot be applied on a day-to-day														
	Outdated guidelines	✓													1
	Guideline trustworthiness (evidence quality, content, developer)	✓		✓	✓	✓	✓				✓				6
Patients	Language and literacy problems				✓										1
	Lack of motivation, compliance and knowledge to follow the recommendations		✓							✓		✓	✓		4
	Patients' financial situation and occupational status							✓	✓						2

Environment	Lack of protocols and processes of dissemination and implementation		✓		✓						✓	✓	✓		5
	Lack of resources	✓			✓	✓	✓				✓	✓	✓		7
	Lack of time	✓		✓			✓		✓	✓		✓	✓		7
	Lack of clinical audit and feedback	✓							✓						2
	Difficulties with availability of medicines or test				✓	✓			✓					✓	4
	Lack o financial incentives	✓					✓								2
	Lack of training								✓	✓			✓		3
	Insurance does not cover recommendations		✓	✓		✓								✓	4
	Lack of regulation and supervision			✓											1
	Lack of evidence-based culture and education			✓											1
	Lack of policy makers supports			✓					✓						2
	Deficiencies in the referral of patients to services				✓										1
